# A rare case of benign retrorectal teratoma in an adult female: Diagnostic challenges and successful surgical management

**DOI:** 10.1016/j.ijscr.2024.110052

**Published:** 2024-07-20

**Authors:** Abdulrahman Shbani, Qamar Suleiman, Fadi Suleiman

**Affiliations:** a Tartous university, Faculty of Medicine, Tartous, Syrian Arab Republic; b Department of General surgery, Tartous University, Tartous, Syrian Arab Republic

**Keywords:** Ovarian teratoma, Retrorectal teratoma, Case report

## Abstract

**Introduction:**

Retrorectal tumors are rare growths with various types which are found in the space behind the rectum. They can be diverse and are often diagnosed through imaging and surgery.

**Presentation:**

A 31-year-old female patient came to the clinic with concerns about irregular periods and constipation, but no history of abdominal pain, pelvic pressure, or weight loss. She had a previous surgery to remove an ovarian teratoma when she was three months old. Physical exams and lab tests showed no significant findings, except for a pelvic ultrasound that revealed a normal right ovary with a small follicle and a missing left ovary due to prior surgery for a dermoid cyst. Another cyst, measuring 8.2 × 9.3 × 5.7 cm, was found behind the uterus, believed to be a presacral cyst possibly originating from elsewhere.

Further investigation with a CT scan confirmed the presence of a large cyst near the rectum, leading to an open surgical procedure to remove it. The cyst, located deep behind the rectum and next to the levator ani muscle, contained a substance resembling cheese with hair, suggesting a benign dermoid cyst with granulation tissue. The surgery was successful, and the diagnosis was confirmed through histopathological analysis.

**Discussion:**

Retrorectal teratomas are rare germ cell tumors that mainly affect children, often presenting with vague symptoms like constipation. Diagnosis involves imaging tests like ultrasound, CT scans, and MRI, with surgical removal being the primary treatment option. Recurrence rates are low with complete excision of benign tumors.

**Conclusion:**

Retrorectal or presacral teratomas are rare tumors with vague symptoms, making diagnosis difficult. They are often detected late and require radiological assessment for surgical planning. Treatment success hinges on a coordinated effort by skilled radiologists and surgeons specializing in pelvic and oncological care to ensure favorable outcomes with lower recurrence rates and risks.

## Highlights

• Teratomas are the primary germ cell tumors found in children can be found in various parts of the body, including the head and neck, mediastinum, intraperitoneal or retroperitoneal areas, and the retrorectal or presacral space.
• Symptoms of benign retrorectal tumors are often vague or related to pressure caused by the mass. They are typically discovered incidentally during pelvic or rectal exams or through imaging for other health concerns.
• The clinical diagnosis of retrorectal teratomas can be done by multiple diagnostic methods like transabdominal ultrasound US, digital rectal examination (DRE), CT scan and MRI. The main treatment for RTs involves completely removing them surgically.

## 1 Introduction

Retrorectal tumors are uncommon growths with a wide variety of histological types, which are typically located within the retrorectal space. This space, also known as the presacral space, is defined by the sacrum posteriorly, the peritoneal reflection superiorly (between the second and third sacral segments), the rectum anteriorly and the levator ani and coccygeus muscles inferiorly. It has the potential to harbor a diverse array of lesions, such as a congenital, osseous, inflammatory, neurogenic and others growths [[3], [4], [5],^8^,^9^].

Due to their position behind the peritoneum, retrorectal tumors often grow significantly before causing symptoms by pressing on nearby organs. Diagnosis of these tumors typically involves a combination of methods like digital rectal examination and radiological tools like transabdominal ultrasound, CT scan, and MRI [[2], [3], [4],^8^,^9^].

In the end, the final diagnosis is determined by surgically removing the tumor, which not only confirms the diagnosis but also acts as a treatment. The chosen surgical method is based on factors such as the tumor's location, size, and its interaction with nearby tissues. In this case, we discuss a case involving a 31-year-old woman with a retrorectal neoplasm that was successfully removed using an anterior approach, with histological analysis revealing a benign dermoid cyst.

The study has been documented according to the SCARE guidelines [11].

## 2 Presentation

A 31-year-old female patient presented to our clinic complaining of menstrual irregularities and constipation. She had no history of abdominal pain, pelvic pressure, or weight loss. Physical examination and lab tests were normal. She had a history of ovarian teratoma removed in infancy when she was 3 months old.

A pelvic ultrasound revealed that the right ovary is normal and contains a follicle measuring 1.5 cm. It was not possible to see the left ovary due to a history of previous surgery for a dermoid cyst.

A cyst was seen behind the uterus measuring 8.2 by 9.3 by 5.7c.m with a turbid liquid inside it. The cyst was noticed with a hyperechoic intensity with no explicit perfusion in it.

**Fig. 1:**
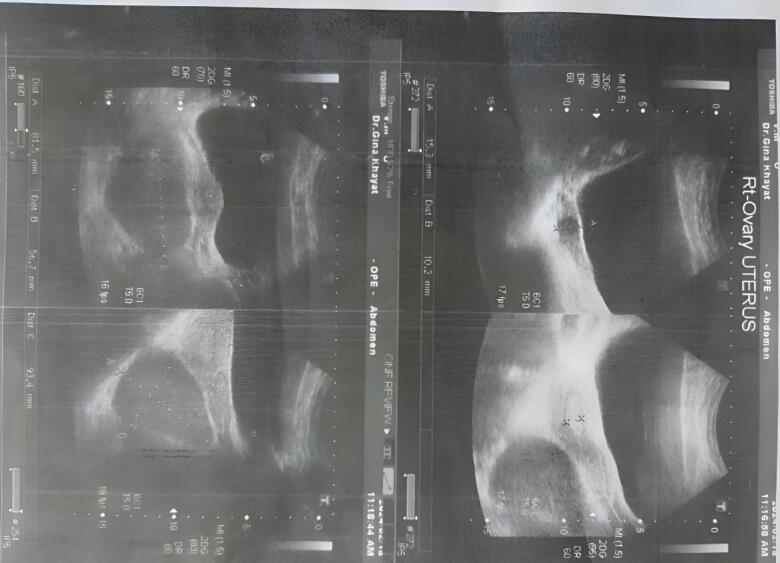
Abdominal ultrasound showing a large cyst behind the cervix.

This cyst was located behind the sigmoid colon, which raised suspicion that it was a presacral cyst and may be of another origin. No free fluid was seen (Fig. 1).

**Figs. 2 and 3:**
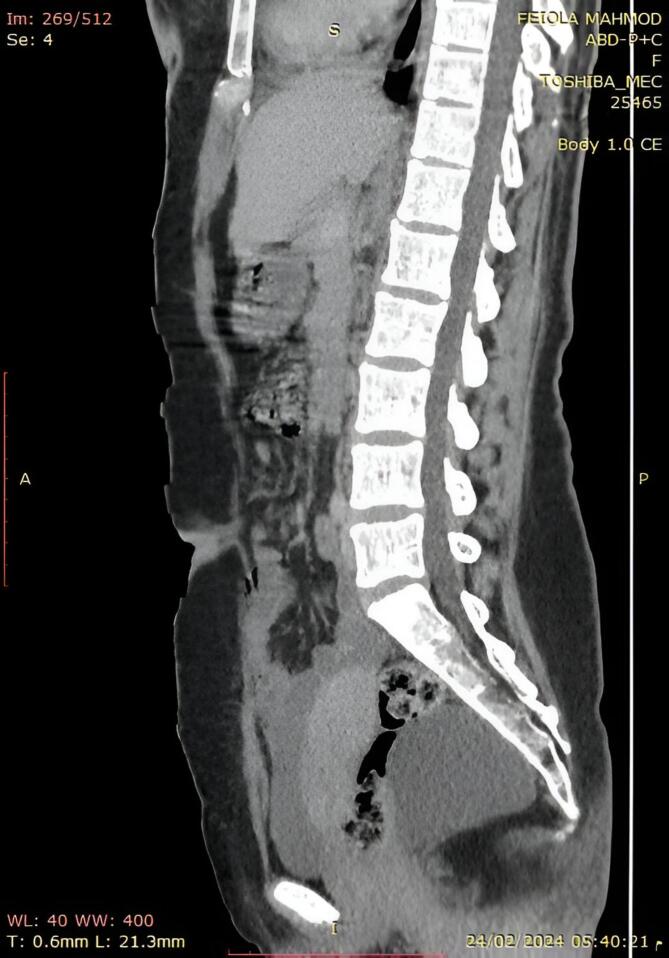

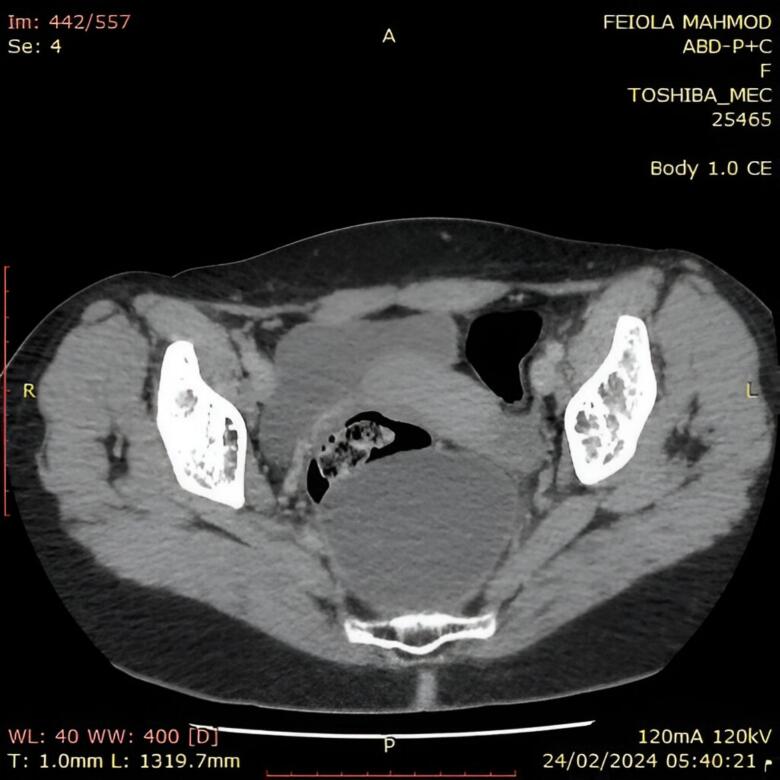
Computed tomography of presacral teratomas. Axial (A) and sagittal (B) sections. “A” Axial image of contrast enhanced computed tomography of abdominal and pelvis showing a large cystic mass near the back edge of the middle and lower portions of the rectum. “B” Sagittal image of abdominal ultrasound showing a large cyst behind the cervix.

CT scan was performed and revealed a sizable cyst measuring 8 by 7 by 5.6 cm, located in the fat near the back edge of the middle and lower portions of the rectum (Figs. 2 and 3).

Based on these findings, general surgeons performed an open surgical procedure under general anesthesia to extract the cyst. A Pfannenstiel incision was performed, revealing the protruding rectum. The pelvic and abdominal cavities were found to be entirely clear. A large, smooth mass was felt deep behind the rectum, situated beneath the pelvic muscles and adjacent to the levator ani. Surgeons attempted to separate the cyst from its surroundings, but it contained a cheese-like material with hair that emerged during dissection. The entire cyst was successfully removed, and a drainage tube was inserted.

**Fig. 4:**
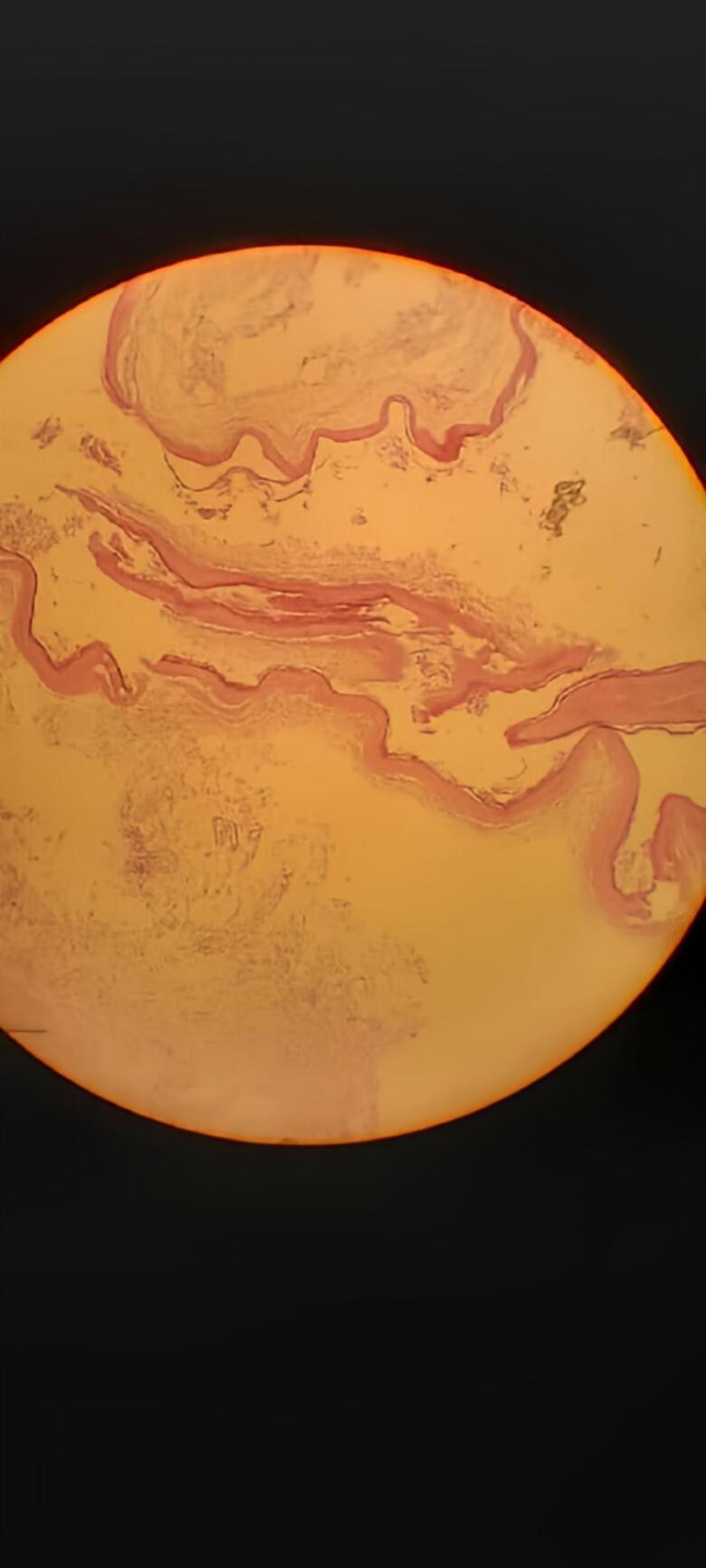
Dermoid cyst with granulation tissue: Photomicrograph shows mature stratified squamous epithelium lining the cyst. Note the skin appendages.

Histopathological examination of cyst samples revealed that it was an elastic cystic wall containing cheese-like substance with hair, confirming it to be a benign dermoid cyst with granulation tissue (Fig. 4).

## 3 Discussion

Teratomas belong to the group of non-seminomatous germ cell tumors. Pluripotent cells develop into ectoderm, mesoderm, and endoderm elements within the teratoma. They can be categorized based on the present layer (mono-, bi-, or tridermal), epithelial lining (epidermoid, dermoid, or teratoid), tissue maturity level (mature or immature), presence of malignant components, and tumor composition (cystic, solid elements, or a combination) [1,4,7,9].

Teratomas can be found in various parts of the body, including the head and neck, mediastinum, intraperitoneal or retroperitoneal areas, and the retrorectal or presacral space. Specifically, 11 % of tumors located in the retroperitoneum are teratomas, with a higher incidence among children [4].

The location of this tumor in our case was near the back edge of the middle and lower portions of the rectum.

Teratomas are more frequently seen in females. They are more prevalent in children compared to adults; however, if detected in adults, they are more likely to be malignant [9]. Unlike our case which was diagnosed as a benign teratoma in an adult female.

Various embryological structures develop in the retrorectal space, giving rise to a diverse range of benign and malignant tumors. The majority of tumors in this area are non-cancerous, with developmental cysts (such as epidermoid, dermoid, and tailgut cysts, as well as benign teratomas) and schwannomas being the most common types. Previous studies have indicated that retrorectal tumors are uncommon, occurring in approximately 1 out of 40,000 admissions. However, as many of these tumors do not cause symptoms, the actual prevalence of these lesions is uncertain [2,4,10].

Among congenital lesions, teratomas are typically located in the sacrococcygeal area and remain the most frequently encountered tumor in infants, with 80 % being detected within the first six months of birth [5].

Symptoms of benign retrorectal tumors are often vague or related to pressure caused by the mass. They are typically discovered incidentally during pelvic or rectal examinations or through imaging for other health concerns. Patients may report lower back or abdominal pain, a feeling of pelvic or rectal pressure, changes in bowel habits like constipation, masses that occasionally obstruct labor in childbearing age women. If invasion of pelvic nerves happens, rectal incontinence, urinary incontinence, and sexual dysfunction may occur [2,4].

Larger tumors can lead to challenges with defecation or even urinary difficulties. Also, infections are common with retrorectal tumors, causing pain and potentially manifesting as abscesses, draining sinuses, or fistulas [2].

In our case, the patient denied any distinctive symptoms except menstrual disturbances and feeling constipated.

The clinical diagnosis of retrorectal teratomas can be done by multiple diagnostic methods like transabdominal ultrasound US, digital rectal examination (DRE), CT scan and MRI. Digital rectal examination (DRE) plays a crucial role in the evaluation of retrorectal tumors (RTs) by providing information on the size, location, consistency, and relationship of the tumor with the rectal wall and surrounding structures. This information is essential for surgical planning as it helps determining the proximal and distal extent of the tumor, guiding the selection of the most appropriate surgical approach [2, 3, 4, 9, 8].

Spiral CT scans and MRI, are valuable tools for accurately identifying and differentiating between benign and malignant tumors and determining the surgical approach in conjunction with the physical examination. A CT scan of the pelvis can detect small tumors, differentiate them from solid or cystic lesions, and show if there is sacral involvement or invasion of nearby structures.

MRI in particular is useful in determining and evaluating soft tissue levels and the presence of bone and nerve invasion [3,4,8].

The patient in our case had undergone to pelvic ultrasound and CT scan which they detect the cyst measurement and its relationship with surrounding tissues.

In a comprehensive literature review of 341 studies including 1708 patients reported that accuracy of MRI and CT scan on specific tissue resection tumor type was only 28 % and 18 %, respectively [3].

This means that the exact diagnosis is not determined until surgical removal is performed, Which we did in our case.

Retrograde mass biopsy of the rectum is a contentious procedure due to the potential risks of contamination or tumor dissemination. It is recommended to only carry out this biopsy if the lesion is not easily detectable and if a pathological diagnosis is necessary for determining adjuvant therapy. Moreover, the histological classification of a retrorectal tumor does not impact the selection of the surgical method. As a result, biopsies are typically not conducted prior to surgery [3,8,9].

In our case, Samples of the cyst were sent to histopathological examination after the surgical procedure, which confirmed the diagnosis.

The main treatment for RTs involves completely removing them surgically by an open surgery procedure or laparoscopic surgery [6,11]. The choice of surgical method depends on factors such as the tumor's location, size, characteristics, and whether it has invaded nearby structures like the sacrum, pelvic sidewalls, rectum, and anal canal. The medical literature describes three primary surgical approaches for removing retrorectal tumors: the anterior approach (open or laparoscopic), the combined abdominoperineal approach, and the perineal or posterior approach [3,4,9,10].

The primary factor that determines the used surgical method, in addition to the surgeon's preference, is the location of the tumor in relation to the S3 vertebra. Typically, tumors above S3 require an anterior or combined approach, while those below S3 are removed using a posterior approach [3,4].

Treatment options such as medical or radiation therapy are not very effective in addressing primary retrorectal lesions. Tumors in this area often do not respond well to chemotherapy or radiation, although radiation may offer some relief in certain cases. On the other hand, metastases to this region, particularly from colorectal cancer, tend to show positive responses to a combination of chemotherapy and radiation therapy [9]. In our case, we did not use them in treating our patient. Moreover, in our case with transabdominal approache, intraoperative rupture of the cyst was expected because of the thin, strong adherence, and the narrow surgical space.

Research has shown that the recurrence rate in cases of primary retrorectal lesions can vary from 0 % to 15 %. Nevertheless, a complete excision of a benign tumor is anticipated to result in a 0 % recurrence rate [4,9].

The unusual combination of a large teratoma tumor in a specific location, presenting with misleading symptoms, being benign in this age, and might be a recurrent tumor from childhood makes this case exceptionally rare and any similar cases in the future deserve to be carefully studied and dealt with.

## 4 Conclusion

Retrorectal or presacral teratomas are uncommon and can present with various non-specific symptoms. They pose a diagnostic challenge and are frequently identified at a later stage. Radiological evaluation plays a crucial role in planning surgical interventions, which are fundamental in treating these tumors. Optimal outcomes, minimal recurrence, and reduced risks can be achieved through a multidisciplinary approach involving experienced radiologists and surgeons specializing in pelvic and oncological procedures.

## Consent

Written informed consent was obtained from the patient for publication and any accompanying images. A copy of the written consent is available for review by the Editor-in-Chief of this journal on request.

## Authorship

All authors attest that they meet the current ICMJE criteria for authorship.

## Ethical approval

Our institution does not require ethical approval for reporting individual cases or case series.

## Funding

The authors received no financial support for the research, authorship, and/or publication of this article.

## Author contribution

**Abdulrahman Shbani is the first author,** contributed to drafting, editing & reviewing. The author reviewed and accepted the paper.

**Qamar Suleiman** contributed to drafting, reviewing, & editing. The author reviewed and accepted the paper.

**Fadi Suleiman is the supervisor,** contributed to editing, reviewing & mentorship. The author reviewed and accepted the paper.

## Guarantor

**Abdulrahman Shbani** accepts full responsibility for the work, had access to the data, and controlled the decision to publish.

## Research registration number

Not Applicable since it is a case report.

## Conflict of interest statement

The authors declare that they have no known competing financial interest or personal relationship that could have appeared to influence the work reported in this paper.
